# Protein structure quality assessment based on the distance profiles of consecutive backbone Cα atoms

**DOI:** 10.12688/f1000research.2-211.v3

**Published:** 2013-12-17

**Authors:** Sandeep Chakraborty, Ravindra Venkatramani, Basuthkar J. Rao, Bjarni Asgeirsson, Abhaya M. Dandekar

**Affiliations:** 1 Department of Biological Sciences, Tata Institute of Fundamental Research, Mumbai, 400 005, India; 2 Department of Chemical Sciences, Tata Institute of Fundamental Research, Mumbai, 400 005, India; 3 Science Institute, Department of Biochemistry, University of Iceland, Reykjavik, IS-107, Iceland; 4 Plant Sciences Department, University of California, Davis, CA 95616, USA

**Keywords:** Computational biology ; protein structure prediction ; Model quality assessment programs ; Boltzmann distribution ; Annsen's thermodynamic hypothesis ; statistical potentials ; protein backbone ; decoy sets ;

## Abstract

Predicting the three dimensional native state structure of a protein from its primary sequence is an unsolved grand challenge in molecular biology. Two main computational approaches have evolved to obtain the structure from the protein sequence - *ab initio/de novo* methods and template-based modeling - both of which typically generate multiple possible native state structures. Model quality assessment programs (MQAP) validate these predicted structures in order to identify the correct native state structure. Here, we propose a MQAP for assessing the quality of protein structures based on the distances of consecutive Cα atoms. We hypothesize that the root-mean-square deviation of the distance of consecutive Cα (RDCC) atoms from the ideal value of 3.8 Å, derived from a statistical analysis of high quality protein structures (top100H database), is minimized in native structures. Based on tests with the top100H set, we propose a RDCC cutoff value of 0.012 Å, above which a structure can be filtered out as a non-native structure. We applied the RDCC discriminator on decoy sets from the Decoys 'R' Us database to show that the native structures in all decoy sets tested have RDCC below the 0.012 Å cutoff. While most decoy sets were either indistinguishable using this discriminator or had very few violations, all the decoy structures in the fisa decoy set were discriminated by applying the RDCC criterion. This highlights the physical non-viability of the fisa decoy set, and possible issues in benchmarking other methods using this set. The source code and manual is made available at https://github.com/sanchak/mqap and permanently available on 10.5281/zenodo.7134.

## Introduction

The structure of a protein is a veritable source of information about its physiological relevance in the cellular context ^1^. In spite of rapid technical advances in crystallization techniques, the number of protein sequences known far exceeds the known structures. There are essentially two different computational approaches to predict protein structures from its primary sequence: 1) Template based methods (TBM) which are based on features obtained from the database of known protein structures ^2– 4^ and 2) *ab initio* or *de novo* methods which are based on the intrinsic laws governing atomic interactions and are applicable in the absence of a template structure with significant sequence homology ^5, 6^. While at present TBM methods fare much better than the *de novo* approaches, the requirement of a known template protein can sometimes be a constraining factor. Both these methods typically generate multiple possibilities for the native structure of a given sequence. Selecting the best candidate from the set of putative structures is an essential aspect that is performed by model quality assessment programs (MQAP).

MQAPs can be classified as energy based, consensus based or knowledge based. The refinement of structures based on modeling of atomic interactions in energy based methods, such as molecular dynamic simulations, are subject to limited sampling of possible conformations due to large run times, and force field inaccuracies due to the approximations involved in describing the dynamics of large multi-atomic systems ^7– 10^. Consensus methods are based on the principle that sub-structures of the native structure are likely to feature frequently in a set of near-native structures ^11– 14^. These methods are currently the best performing amongst MQAPs ^13^, but are prone to be computationally intensive due structure-to-structure comparison of all models ^14^, and are of limited use when the number of possible structures is small ^15^. Knowledge based methods rely on the assignment of an empirical potential (also known as statistical potential) from the frequency of residue contacts in the known structures of native proteins ^16, 17^. In statistical physics, for a system in thermodynamic equilibrium, the accessible states are populated with a frequency which depends on the free energy of the state and is given by the Boltzmann distribution. The Boltzmann hypothesis states that if the database of known native protein structures is assumed to be a statistical system in thermodynamic equilibrium, specific structural features would be populated based on the free energy of the protein conformational state. Sippl argued using a converse logic that the frequencies of occurrence of structural features such as interatomic distances in the database of known protein structures could determine a free energy (potential of mean force) for a given protein conformation, and thus be used to discriminate the native structure ^18, 19^. A crucial aspect in applying statistical potentials is the proper characterization of the reference state ^20^. The application of such empirical energy functions to predict and assess protein structures, while quite popular, are vigorously debated ^21, 22^, and several approaches for using statistical potentials for protein structure prediction been described to date ^20, 23– 26^.

Here, we propose a new statistical potential based MQAP for assessing the quality of protein structures based on the distances of consecutive C *α* atoms - Protein structure quality assessing based on Distance profile of backbone atoms (PROQUAD). We first propose a statistical potential based on the distance of consecutive C *α* distances. In a set of high quality protein structures (top100H ^27^), we demonstrate that the distance between consecutive C *α* atoms are distributed normally with a mean of 3.8 Å and standard deviation of 0.04 Å. Based on this observation, the reference state for our statistical potential calculations is defined as one where all consecutive C *α* atoms are 3.8 Å apart. We propose a scoring function which measures the deviation of consecutive C *α* atoms from 3.8 Å, and hypothesize that this score is minimized in native structures. Based on the top100H database, we chose a cutoff of 0.012 Å for this scoring function to identify non-native states. We show that all the decoy structures from the fisa decoy set taken from the Decoys 'R' Us database ^28^ are distinguished using this discriminator. It has been previously proposed that native structures have constrained interatomic distances ^29^. Interatomic distances, and other metrics, have been combined in several such methods - Molprobity ( http://molprobity.biochem.duke.edu/), PROSA ( https://prosa.services.came.sbg.ac.at/prosa.php) and the WHATIF server ( http://swift.cmbi.ru.nl/whatif) ^30– 32^. These identify possible anomalies in a given protein structure. While Molprobity and WHATIF identified steric clashes in the decoy structure in fisa, distance checks between consecutive C *α* are not part of checks in these methods, and they failed to detect the consecutive C *α* atoms anomaly in the fisa decoy set.

Thus, we propose a simple and fast discriminator for protein structure quality based on the distance profiles of consecutive backbone C *α* atoms that identifies decoy structures that are physically nonviable.

## Results and discussion

**Figure 1.**
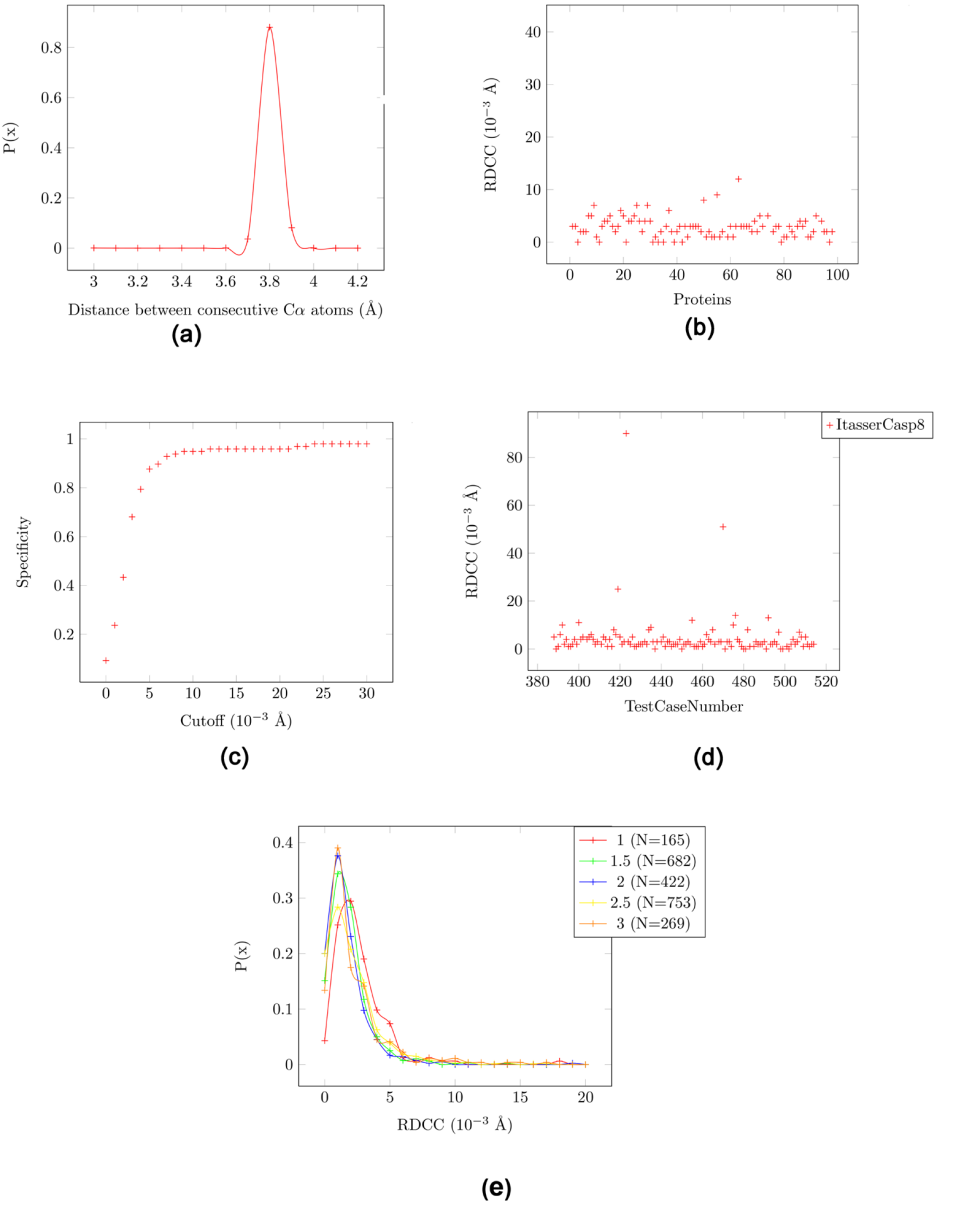
Root-mean-square deviation of the distance of consecutive C *α* (RDCC) atoms from the ideal value of 3.8 Å. ( **a**) Probability distribution (P(x)) for the distance of consecutive C *α* in ~100 proteins in the top100H database. ( **b**) RDCC in ~100 high quality structures from the top100H database. ( **c**) Variation in specificity based on the cutoff value. We choose 0.012 Å as the cutoff for filtering out non-native structures. ( **d**) RDCC in I-TASSER CASP8 decoy suite. ( **e**) RDCC for protein structures based on the resolution.

The frequency distribution of the distance of consecutive C *α* atoms in ~100 proteins in the top100H database (a database consisting of high quality structures) ^27^ shows that the distance between consecutive C *α* atoms are distributed normally with a mean of 3.8 Å and standard deviation of 0.04 Å ( Figure 1a). Out of 16,162 pairs of consecutive C *α* atom distances, 14,281 (88%) were spaced 3.8 Å apart, 1297 (8%) were spaced 3.9 Å apart and 553 (3%) were spaced 3.7 Å apart. Only 31 (0.1%) pairs of consecutive C *α* atom distances had values different than these (highest being 4 Å and the lowest being 2.9 Å). It would be interesting to correlate these distance deviant residue pairs to structural or functional aspects of the protein - *It is well worth examining every outlier and either correcting it if possible, giving up gracefully if it really cant be improved (more often true at low resolution), or celebrating the significance of why it is being held in an unfavorable conformation* ^33^.

The *cis* confirmations of peptide bonds are mostly responsible for these deviations. For example, in the protein concanavalin B (PDBID:1CNV), there are four violation of the 3.8 Å constraint: Ile33/Ser34 - 4 Å, Ser34/Phe35 - 3 Å, Pro56/Ser57 - 4 Å and Trp265/Asn266 - 3.4 Å. These all these deviations are noted in the PDB file as footnotes, mentioning that ‘peptide bond deviates significantly from *trans* conformation’ ^34^. Another example is the Glu223-Asp24 violation in PBDid:1ADS, which is between two *cis* prolines (as noted in the PDB file) ^35^. However, these conformations are rare and not expected to occur frequently in a protein structure.

Figure 1b plots the root-mean-square deviation of the distance of consecutive C *α* (RDCC) for these ~100 proteins. All structures in the top100H database have low RDCC values, barring three proteins (PDBids: 2ER7, 1XSO and 4PTP), which had multiple conformations for some residues, and were excluded from the processing. This validates our hypothesis that RDCC is minimized in native structures. Hence, structures that have a RDCC value more than a user specified threshold can be pruned out as structures with low quality or non-native structures.

We evaluated the results using the measures of specificity (the ability of a test to identify negative results) which is defined as:

$$specificity=\frac{TN}{FP+TN}\;\;\;\;\;(1)$$

(TN = true negatives, FP = false positives). The specificity variation with the cutoff chosen is shown in Figure 1c. We choose 0.012 Å as the cutoff value for RDCC, which has a specificity of 1. We also plot the RDCC of the 121 testcases ( Figure 1d) in the I-TASSER decoy set - http://zhanglab.ccmb.med.umich.edu/casp8/decoys ^36^. Only five sets have RDCC values above the 0.012 Å threshold: T0492 - 0.013 Å, T0476 - 0.014 Å, T0419 - 0.025 Å, T0470 - 0.051 Å, T0423 - 0.09 Å. Some of these are the result of erroneous residue numbering in the CASP8 I-TASSER decoy set. For example, Ala24 is mistakenly numbered as Ala19 in T0423 (PDBid:3D01, identified by doing a BLAST search). Correcting this numbering results in a RDCC of 0.002 Å. Similarly, T0470 (PDBid:3DJB) has a correct RDCC of 0.001 Å, since Ser112 is mistakenly numbered as Ser101.

Figure 1e plots the frequency distribution of RDCC values of protein structures based on their resolution. The RDCC values are much lower than the 0.012 Å cutoff proposed. The non homologous structures (20% identity cutoff) are obtained from the PISCES database ( http://dunbrack.fccc.edu/PISCES.php). Certain outliers have been removed - for example, PDBid:2JLI mentions a ‘cleaved peptide bond between N263 and P264’. The distance between the C *α* atoms of N263 and P264 in this protein is 9.4 Å. Table 1 shows the mean and standard deviation for these sets, and demonstrates that the RDCC values are independent of the resolution of the structure under consideration.

**Table 1.** Mean and standard deviation (SD) of RDCC values for structures based on resolution. The number N signifies the number of protein structures analzyed that have resolution less than the specified number, but more than the previous one. For example, there are 165 protein with less than 1 Å resolution, and 682 proteins which have more than 1 Å but less than 1.5 Å resolution, and so on.

Resolution(Å)	N Mean	RDCC(10-3 Å)	SD(10-3 Å)
1.0	165	3.0	4.3
1.5	682	1.9	2.7
2.5	422	2.2	2.6
2.0	753	1.8	2.8
3.0	269	2.2	2.5

**Figure 2.**
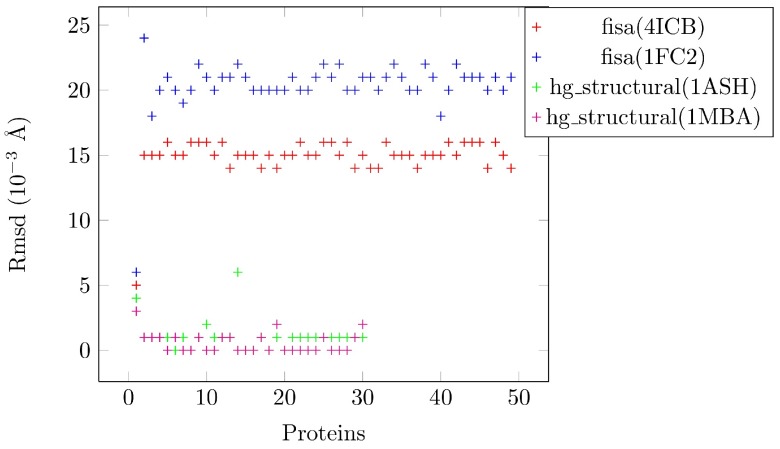
Root-mean-square deviation (RMSD) of the distance of consecutive C *α* (RDCC) atoms from the ideal value of 3.8 Å in decoy sets. The hg_structal and misfold decoy sets are indistinguishable using the distance discriminator, unlike the fisa decoy set. We have shown ~25 decoy structures from the fisa set, but the values apply to all the decoys (more than 500). The first protein (the native structure) in each set has RDCC below the 0.012 Å cutoff.

**Figure 3.**
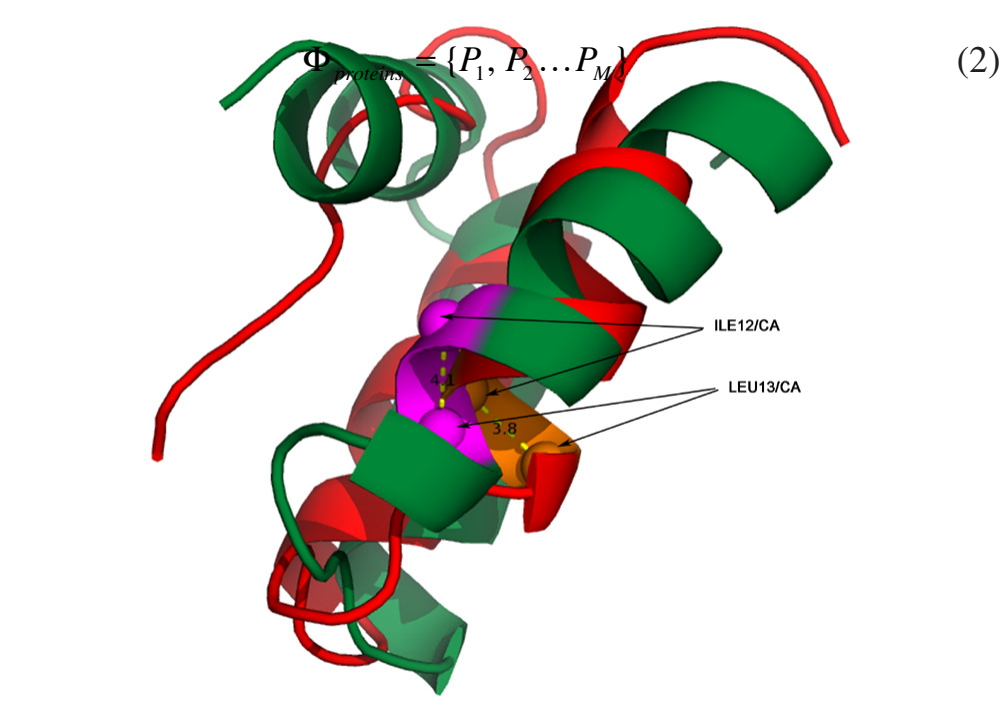
Superimposition of the native structure and a decoy structure (AXPROA00-MIN) for a protein (PDBid:1FC2) taken from the fisa decoy set. The native structure is in red, and the decoy structure is in green. The structures are superimposed using MUSTANG ^58^. The distance between Ile12/C *α* and Leu13/C *α* atoms is 3.8 Å and 4.1 Å in the native and the decoy structures, respectively.

We have applied this cutoff on decoy sets from the Decoys 'R' Us database ^28^. The first protein (the native structure) in all decoy sets has RDCC below the 0.012 Å cutoff ( Figure 1c). Figure 2 shows the RDCC for the hg_structral and fisa decoy sets from the Decoys 'R' Us database. All 500 decoy structures in each protein structure in the fisa decoy set are discriminated by applying the RDCC criterion. Figure 3 shows the superimposition of the native structure and the first decoy structure (AXPROA00-MIN) for a protein (PDBid:1FC2) taken from the fisa decoy set. The distance between Ile12/C *α* and Leu13/C *α* atoms is 3.8 Å and 4.1 Å in the native and the decoy structures, respectively. According to our hypothesis, a 4.1 Å distance between consecutive C *α* atoms is typically unfeasible in protein structures, and their occurrence should be relatively rare. The presence of such deviations throughout the protein structures categorizes it as a non-native structure. MolProbity ^30^ and ProSA ^31^ are two programs used as a pre-processing step for structures used in CASP ^38^. MolProbity was able to discriminate the decoy structure (AXPROA00-MIN) from the native structure (PDBid:1FC2) using a metric called the ClashScore (the number of serious steric overlaps) and the C *β* deviations ^39^. PROSA was unable to discriminate between the decoy and the native structures, reporting equivalent Zscores of -4.12 and -5.28, respectively. The WHATIF server report also reports steric clashes in the decoy structures ( Data File 1). None of the above mentioned methods use a metric similar to the RDCC proposed in this paper, and thus did not report the abnormal distance between consecutive C *α* atoms in the decoy structure.

The hg_structal and misfold decoy sets are indistinguishable using this distance discriminator, while only a few decoy structures failed in the 4state_reduced decoy set. This relationship between RDCC and proteins structure quality is therefore not an equivalence relationship. In propositional calculus, a relationship is equivalent if 'A' implies 'B' and 'B' implies 'A'. A high RDCC implies a low quality structure, but a low quality structure does not necessitate a high RDCC. We therefore suggest the usage of the RDCC measure as a first pass to rule out the non-native contacts prior to applying other discriminators.

The model quality assessment program (MQAP) used to choose the best structure from the multiple closely related structures generated by structure prediction programs is of critical importance. We have in the past used electrostatic congruence to detect a promiscuous serine protease scaffold in alkaline phosphatases ^40^ and a phosphoinositide-specific phospholipase C from *Bacillus cereus* ^41^, and a scaffold recognizing a *β*-lactam (imipenem) in a cold-active *Vibrio* alkaline phosphatase ^42, 43^. However, continuum models ^44^ that compute potential differences and pK *_a_* values from charge interactions in proteins ^45^ are sensitive to the spatial arrangement of the atoms in the structure. Thus, an incorrect model will generate an inaccurate electrostatic profile of the peptide ^46^. It is thus possible to functionally characterize a protein from its sequence by applying such *in silico* tools subsequent to the protein structure prediction and MQAPs tools ^47^.

The estimation of the model quality by MQAPs is achieved by formalizing a scoring function ^48^, referred to as a knowledge-based or statistical potential, constructed from the database of known structures, assuming that the distribution of the structural features obtained from these structures follows the Boltzmann distribution ^20, 23, 24, 26^. The validity of statistical potential and the method to choose a proper reference frame in such models are still widely debated ^21, 22^. Methods that use consensus values from numerous models outperform other MQAP methods ^11– 14^, and are ‘very useful for structural meta-predictors’ ^49^. It has been shown that many of the MQAP programs perform considerably better when different statistical metrics are combined ^50– 53^. The state of the art methods for predicting structures ^54^ and MQAPs ^38, 49, 55^ are evaluated by researchers every two years.

Here, we propose a discriminator (RDCC) based on the distance of consecutive C *α* atoms in the peptide structure. The discriminator is independent of the database of structures ^56^, and is thus an absolute discriminator. Our proposed RDCC criterion is satisfied in high quality protein structures taken from the top100H database. As a specific application, we show that all decoy structures in the fisa decoy set from the Decoys 'R' Us database C *α* atoms do not satisfy this criterion. It has been previously shown that the fisa decoy set violates the van der Waals term ^57^. We propose a fast complementary method to identify this transgression. It is also an interesting fact that most consensus methods will fare poorly in the fisa decoy set, since the majority of sub-structures are incorrect in all the decoy sets. Therefore, the fisa decoy set consists of physically nonviable structures and one should exercise caution when benchmarking other methods using this decoy set ^58^.

Data File 1: WHATIF server report The WHATIF server report for the AXPROA00-MIN decoy structure. Note that it did not identify the anomalous C-alpha atom distances in this protein.

## Materials and methods

The set of proteins Φ *_proteins_* consists of the native structure *P*_1_ and M-1 decoys structures ( Equation 2). We ignore the first x= *IgnoreNTerm* and last y= *IgnoreCTerm* pairs of residues in the protein structure to exclude the terminals ( Equation 3). For every consecutive pair of residues in the structure we calculate the distance between the consecutive C *α* atoms ( *Res _n_*( *Cα*) and *Res*_n+1_( *Cα*)), and its deviation from the ideal value of 3.8 Å. The square of the summation of these deviations is then normalized based on the number of pairs processed, and results in the *CADistScore.* We hypothesize that *CADistScore ^P^* ^1^ is minimum in a native structure ( Equation 4). Algorithm 1 shows the pseudocode for the function that generates the *CADistScore*.

**Algorithm 1: AssessCADist()**

**Input**: *P*_1_ : Protein under consideration

**Input**: *IgnoreNTerm*: Ignore this number of residues in the N Terminal

**Input**: *IgnoreCTerm*: Ignore this number of residues in the C Terminal

**Output**: *CADistScore*: Score indicating deviation of successive C *α* atoms from 3.8 Å

**begin**

    
*CADistScore* = 0 ; *NumberCompared* = 0 ; *N* = NumberOfResidues( *P*_1_);

    
**for** *p* ← *IgnoreNTerm* **to** *N – IgnoreCTerm* **do**

        
*q* = *p* + 1 ;

        
*CADist* = Distance( *p, q, Cα, Cα*)

        
*NumberCompared* = *NumberCompared* + 1 ;

        
*diff* = absolute( *CADist* – 3.8 *Å*) ;

        
*CADistScore* = *CADistScore* + *diff* * *diff*;

    
**end**

    /* Normalize */

    
*CADistScore* = *sqrt*( *CADistScore/*( NumberCompared * NumberCompared));

    
**return** ( *CADistScore*);

**end**

$$\Phi_{proteins}=\{P_{1},P_{2}\ldots P_{M}\}\;\;\;\;\;\;\;\;\;\;\;\;\;\;\;\;\;\;\;\;\;\;\;\;\;\;(2)$$

$$CADistScore^{P_{i}}=\sqrt{\frac{{\sum {}_{n=1+x}^{N-y-1}(dist(Res}_{n}(C\alpha ),Res_{n+1}(C\alpha ))-3.8){}_{}^{2}}{{\left(N-y-x-2\right)}^{2}}}\;\;\;(3)$$

$$\left[\forall i=2\ldots M](CADistScore^{P1}<CADistScore^{pi})\;\;\;\;\;\;\;\;\;\;\;\;\;\;(4\right)$$

In order to validate our hypothesis on known structures, we applied our discriminator to the top100H database (a database consisting of high quality structures) ^27^ - http://kinemage.biochem.duke.edu/databases/top100.php. In order to benchmark model quality assessment programs, we used decoy sets from the Decoys 'R' Us database ^28^ - http://dd.compbio.washington.edu/. Each set has several structures that are supposed to be ranked worse than the native structure.

Structural superimposition has been done using MUSTANG ^59^. Protein structures were rendered by PyMol ( http://www.pymol.org/). The source code and manual is made available at https://github.com/sanchak/mqap.
