# Supplementary material for: Protein structure quality assessment based on the distance profiles of consecutive backbone Cα atoms
Source: F1000Res. 2013 Dec 17;2:211. Originally published 2013 Oct 10. [Version 3] doi: 10.12688/f1000research.2-211.v3 (PMC3892923; doi:10.12688/f1000research.2-211.v3)
Supplement: Data File 1: WHATIF server report — The WHATIF server report for the AXPROA00-MIN decoy structure. Note that it did not identify the anomalous C-alpha atom distances in this protein. [file f1000research-2-3328-s0000.tgz › Report.pdf]

```
*******************************************************************
********** REPORT OF PROTEIN ANALYSIS  by the WHAT IF program **********
*******************************************************************
```

Date : 2013-08-29
This report was created by WHAT IF version 20130829-1615

This document is a WHAT_CHECK-report that holds the findings of the WHAT IF
program during the analysis of a PDB-file. Each reported fact has an assigned
severity, one of:

```
error  : Items marked as errors are considered severe problems requiring
         immediate attention.
warning: Either less severe problems or uncommon structural features. These
         still need special attention.
note   : Statistical values, plots, or other verbose results of tests and
         analyses that have been performed.
```

If alternate conformations are present, only the first is evaluated. Hydrogen
atoms are only included if explicitly requested, and even then they are not
used in all checks. The software functions less well for non-canonical amino
acids and exotic ligands than for the 20 canonical residues and canonical
nucleic acids.

Some remarks regarding the output:

Residues/atoms in tables are normally given in a few parts:

A number. This is the internal sequence number of the residue used by WHAT IF.
The first residues in the file get number 1, 2, etc.
The residue type. Normally this is a three letter amino acid type.
The sequence number, between brackets. This is the residue number as it was given in the input file. It can be followed by the insertion code.
The chain identifier. A single character. If no chain identifier was given in the input file, this will be a minus sign or a blank.
A model number. If no model number exists, like in most X-ray files, this will be a blank or occasionally a minus sign.
In case an atom is part of the output, the atom will be listed using the PDB nomenclature for type and identifier.

To indicate the normality of a score, the score may be expressed as a Z-value or Z-score. This is just the number of standard deviations that the score deviates from the expected value. A property of Z-values is that the root-mean-square of a group of Z-values (the RMS Z-value) is expected to be 1.0. Z-values above 4.0 and below -4.0 are very uncommon. If a Z-score is used in WHAT IF, the accompanying text will explain how the expected value and standard deviation were obtained.
The names of nucleic acids are DGUA, DTHY, OCYT, OADE, etc. The first character is a D or O for DNA or RNA respectively. This circumvents ambiguities in the many old PDB files in which DNA and RNA were both called A, C, G, and T.

```
cp /home/vriend/whatif/dbdata/supertab.sty .
Atoms without Mendeleev symbol:     353

====================================================
=====================
==== Compound code /home/whatif/httpd/htdocs/server
s/tmp//tmpYJLeqS/====L1.fil
====================================================
=====================

# 1 # Error: Missing unit cell information
No SCALE matrix is given in the PDB file.


# 2 # Error: Missing symmetry information
Problem: No CRYST1 card is given in the PDB file.


SYMMETRY will be unavailable for this molecule.


# 3 # Note: No strange inter-chain connections dete
cted
No covalent bonds have been detected between molecu
les with non-identical
chain identifiers.


# 4 # Note: No duplicate atom names in ligands
All atom names in ligands (if any) seem adequately
unique.


# 5 # Note: In all cases the primary alternate atom
 was used
WHAT CHECK saw no need to make any alternate atom c
orrections (which means
they are all correct, or there are none).


# 6 # Note: No residues detected inside ligands
```

Either this structure does not contain ligands with
 amino acid groups inside
it, or their naming is proper (enough).

7 # Note: No attached groups interfere with hydro
gen bond calculations
It seems there are no sugars, lipids, etc., bound (
or very close) to atoms
that otherwise could form hydrogen bonds.

8 # Note: No probable side chain atoms with zero
occupancy detected.
Either there are no side chain atoms with zero occu
pancy, or the side chain
atoms with zero occupancy were not present in the i
nput PDB file (in which
case they are listed as missing atoms), or their po
sitions are sufficiently
improbable to warrant a zero occupancy.

9 # Note: No probable backbone atoms with zero oc
cupancy detected.
Either there are no backbone atoms with zero occupa
ncy, or the backbone
atoms with zero occupancy were not present in the i
nput PDB file (in
which case they are listed as missing atoms), or th
eir positions are
sufficiently improbable to warrant a zero occupancy
.

10 # Note: All residues have a complete backbone.

No residues have missing backbone atoms.

11 # Note: No C-alpha only residues
There are no residues that consist of only an alpha

carbon atom.

12 # Note: Non-canonical residues
WHAT CHECK has not detected any non-canonical resid
ue(s).

13 # Note: Content of the PDB file as interpreted
 by WHAT CHECK
Content of the PDB file as interpreted by WHAT CHEC
K.
WHAT CHECK has read your PDB file, and stored it in
ternally in what is called
'the soup'. The content of this soup is listed here
. An extensive explanation
of all frequently used WHAT CHECK output formats ca
n be found at
swift.cmbi.ru.nl. Look under output formats. A cour
se on reading this
'Molecules' table is part of the WHAT CHECK website
.

```
     1     1 (    1)    43 (   43) A Protein
      checkset
```

14 # Note: Some notes regarding the PDB file cont
ents
The numbers and remarks listed below have no explic
it validation purpose,
they are merely meant for the crystallographer or N
MR spectroscopists to
perhaps pinpoint something unexpected. See the WHAT
 CHECK course [REF] for
an explanation of terms like 'poor', 'missing', etc
etera (in case those
words pop up in the lines underneath this message).

The total number of amino acids found is 43.

15 # Note: Ramachandran plot
In this Ramachandran plot x-signs represent glycine
s, squares represent
prolines, and plus-signs represent the other residu
es. If too many
plus-signs fall outside the contoured areas then th
e molecule is poorly
refined (or worse). Proline can only occur in the n
arrow region around
phi=-60 that also falls within the other contour is
lands.

In a colour picture, the residues that are part of
a helix are shown in blue,
strand residues in red. Preferred regions for helic
al residues are drawn in
blue, for strand residues in red, and for all other
residues in green. A full
explanation of the Ramachandran plot together with
a series of examples can
be found at the WHAT CHECK website [REF].

In the TeX file, a plot has been inserted here

Chain identifier: A

16 # Note: Secondary structure
This is the secondary structure according to DSSP.
Only helix (H), overwound
or 3/10-helix (3), strand (S), turn (T) and coil (b
lank) are shown [REF].
All DSSP related information can be found at swift.
cmbi.ru.nl/gv/dssp/
This is not really a structure validation option, b
ut a very scattered
secondary structure (i.e. many strands of only a fe
w residues length, many
Ts inside helices, etc) tends to indicate a poor st
ructure. A full
explanation of the DSSP secondary structure determi

nation program together
with a series of examples can be found at the WHAT CHECK website [REF].

```
 Secondary structure assignment
                     10        20        30
40
                      |         |         |
 |
    1 -   43 FNKEQQNAFYEILHLPNLNEEQRNGFIQSLKDDPSQSA
NLLAE
(   1)-(  43)  HHHHHHHHHHHTTTT  HHHHHHHHTHHHH HHHHH
HHHH
```

17 # Note: No rounded coordinates detected
No significant rounding of atom coordinates has been detected.

18 # Note: No artificial side chains detected
No artificial side-chain positions characterized by chi-1=0.0 or chi-1=180.0
have been detected.

19 # Note: No missing atoms detected in residues
All expected atoms are present in residues. This validation option has not
looked at 'things' that can or should be attached to the elemantary building
blocks (amino acids, nucleotides). Even the C-terminal oxygens are treated
separately.

20 # Note: All B-factors fall in the range 0.0 - 100.0

All B-factors are larger than zero, and none are ob
served above 100.0.

21 # Note: No C-terminal nitrogen detected
The PDB indicates that a residue is not the true C-
terminus by including
only the backbone N of the next residue. This has n
ot been observed in this
PDB file.

22 # Note: C-terminus capping
The residues listed in the table below either are p
seudo C-terminal residues,
or have two groups attached of which neither is the
 normal C-terminal O.
In this table REAL means that the C-terminal residu
e is likely to be the real
C-terminus of its chain; OX means that an incorrect
 second oxygen (OXT) was
detected that should not be there; -O indicates tha
t the 'normal' oxygen
(i.e. not the OXT) is missing; OT indicates the det
ection of any other
capping group. C-terminal nitrogen atoms, if any, h
ave already been dealt
with in a previous check and are indicated here by
-N. PSEUDO means that
this is the last visible residue in the chain, but
not the real C-terminus,
i.e. all residues after this one are missing in thi
s chain. BREAK means that
this is the last residue before a chain-break, i.e.
 the chain continues
but after this residue a number of residues is miss
ing. In case a break is
observed the number of residues that seems to be mi
ssing is shown in
brackets. OK means that given the status (REAL, PSE
UDO, BREAK), no problems
were found.

Be aware that we cannot easily see the difference between these errors and
errors in the chain and residue numbering schemes. So do not blindly trust
the table below.

```
   43 GLU  (  43-) A  -        : Unknown problem
```

23 # Note: No OXT found in the middle of chains
No OXT groups were found in the middle of protein chains.

24 # Note: Weights administratively correct
All atomic occupancy factors ('weights') fall in the 0.0-1.0 range, which
makes them administratively correct.

25 # Note: Normal distribution of occupancy values

The distribution of the occupancy values in this file seems 'normal'.

Be aware that this evaluation is merely the result of comparing this file
with about 500 well-refined high-resolution files in the PDB. If this file
has much higher or much lower resolution than the PDB files used
in WHAT CHECK's training set, non-normal values might very well be perfectly
fine, or normal values might actually be not so normal. So, this check is
actually more an indicator and certainly not a check in which I have great
confidence.

26 # Note: All occupancies seem to add up to 0.0 - 1.0.
In principle, the occupancy of all alternates of one atom should add up till
0.0 - 1.0. 0.0 is used for the missing atom (i.e. an atom not seen in the
electron density). Obviously, there is nothing terribly wrong when a few
occupancies add up to a bit more than 1.0, because the mathematics of
refinement allow for that. However, if it happens often, it seems worth
evaluating this in light of the refinement protocol used.

27 # Warning: What type of B-factor?
WHAT CHECK does not yet know well how to cope with B-factors in case TLS has
been used. It simply assumes that the B-factor listed on the ATOM and HETATM
cards are the total B-factors. When TLS refinement is used that assumption
sometimes is not correct. TLS seems not mentioned in the header of the PDB
file. But anyway, if WHAT CHECK complains about your B-factors, and you think
that they are OK, then check for TLS related B-factor problems first.

Temperature not mentioned in PDB file. This most likely means
that the temperature record is absent.
Room temperature assumed

28 # Note: Insufficient residues for statistics
Not enough (intact) amino acids were observed to reliably evaluate the
percentage of buried residues with a low B-factor as a function of the
temperature during X-ray data collection.

Number of (intact) amino acids observed :   43

29 # Note: Number of buried atoms with low B-fact
or is OK
For protein structures determined at room temperatu
re, no more than about 1
percent of the B factors of buried atoms is below 5
.0.

Percentage of buried atoms with B less than 5 :   0
.00

30 # Note: B-factor distribution normal
The distribution of B-factors within residues is wi
thin expected ranges.
A value over 1.5 here would mean that the B-factors
 show signs of
over-refinement.

RMS Z-score : 0.387 over   317 bonds
Average difference in B over a bond :   0.00
RMS difference in B over a bond :   0.00

31 # Warning: B-factor plot useless
All average B-factors are equal. Plot suppressed.

 Chain identifier: A

32 # Note: Introduction to the nomenclature secti
on.
Nomenclature problems seem, at first, rather unimpo
rtant. After all who
cares if we call the delta atoms in leucine delta2
and delta1 rather than
the other way around. Chemically speaking that is c
orrect. But structures
have not been solved and deposited just for chemist

s to look at them. Most
times a structure is used, it is by software in a b
ioinformatics lab. And
if they compare structures in which the one used C
delta1 and delta2 and the
other uses C delta2 and delta1, then that compariso
n will fail. Also, we
recalculate all structures every so many years to m
ake sure that everybody
always can get access to the best coordinates that
can be obtained from
the (your?) experimental data. These recalculations
 will be troublesome if
there are nomenclature problems.

Several nomenclature problems actually are worse th
an that. At the
WHAT CHECK website [REF] you can get an overview of
 the importance of all
nomenclature problems that we list.

33 # Note: Valine nomenclature OK
No errors were detected in valine nomenclature.

34 # Note: Threonine nomenclature OK
No errors were detected in threonine nomenclature.

35 # Note: Isoleucine nomenclature OK
No errors were detected in isoleucine nomenclature.

36 # Note: Leucine nomenclature OK
No errors were detected in leucine nomenclature.

37 # Warning: Arginine nomenclature problem
The arginine residues listed in the table below hav
e their N-H-1 and N-H-2

swapped.

```
   23 ARG  (  23-) A  -
```

38 # Note: Tyrosine torsion conventions OK
No errors were detected in tyrosine torsion angle c
onventions.

39 # Warning: Phenylalanine convention problem
The phenylalanine residues listed in the table belo
w have their chi-2 not
between -90.0 and 90.0.

```
    1 PHE  (   1-) A  -
    9 PHE  (   9-) A  -
   26 PHE  (  26-) A  -
```

40 # Note: Aspartic acid torsion conventions OK
No errors were detected in aspartic acid torsion an
gle conventions.

41 # Warning: Glutamic acid convention problem
The glutamic acid residues listed in the table belo
w have their chi-3
outside the -90.0 to 90.0 range, or their proton on
 OE1 instead of OE2.

```
    4 GLU  (   4-) A  -
   11 GLU  (  11-) A  -
   20 GLU  (  20-) A  -
   21 GLU  (  21-) A  -
   43 GLU  (  43-) A  -
```

42 # Note: Phosphate group names OK in DNA/RNA
No errors were detected in nucleic acid phosphate g
roup naming conventions.

43 # Note: Heavy atom naming OK
No errors were detected in the atom names for non-hydrogen atoms. Please
be aware that the PDB wants us to deliberately make some nomenclature errors;
especially in non-canonical amino acids.

44 # Note: No decreasing residue numbers
All residue numbers are strictly increasing within each chain.

45 # Warning: Unusual bond lengths
The bond lengths listed in the table below were found to deviate more than 4
sigma from standard bond lengths (both standard values and sigmas for amino
acid residues have been taken from Engh and Huber [REF], for DNA they were
taken from Parkinson et al [REF]). In the table below for each unusual bond
the bond length and the number of standard deviations it differs from the
normal value is given.

Atom names starting with "-" belong to the previous residue in the chain. If
the second atom name is "-SG*", the disulphide bridge has a deviating length.

```
    9 PHE  (   9-) A  -    CG   CD1   1.62   11.2
    9 PHE  (   9-) A  -    CG   CD2   1.63   11.7
    9 PHE  (   9-) A  -    CD1  CE1   1.58    6.5
    9 PHE  (   9-) A  -    CD2  CE2   1.64    8.6
    9 PHE  (   9-) A  -    CE1  CZ    1.60    7.2
    9 PHE  (   9-) A  -    CE2  CZ    2.81   47.6
   26 PHE  (  26-) A  -    CG   CD1   1.62   11.2
   26 PHE  (  26-) A  -    CG   CD2   1.62   11.3
   26 PHE  (  26-) A  -    CD1  CE1   1.56    5.9
   26 PHE  (  26-) A  -    CD2  CE2   1.61    7.5
```

```
   26 PHE  (  26-) A  -    CE1  CZ    2.77   46.4
   26 PHE  (  26-) A  -    CE2  CZ    1.63    8.3
```

46 # Warning: High bond length deviations
Bond lengths were found to deviate more than normal from the mean standard
bond lengths (standard values for protein residues were taken from Engh and
Huber [REF], for DNA/RNA these values were taken from Parkinson et al [REF]).
The RMS Z-score given below is expected to be near 1.0 for a normally
restrained data set. The fact that it is higher than 1.5 in this structure
might indicate that the restraints used in the refinement were not strong
enough. This will also occur if a different bond length dictionary is used.

```
 RMS Z-score for bond lengths: 1.762
 RMS-deviation in bond distances: 0.043
```

47 # Warning: Directionality in bond lengths and no X-ray cell
Comparison of bond distances with Engh and Huber [REF] standard values for
protein residues and Parkinson et al [REF] standard values for DNA/RNA shows
a significant systematic deviation.

You have most probably seen symmetry problems earlier. Please correct these
and rerun this check to see the possible implications on the X-ray cell axes.

48 # Warning: Unusual bond angles
The bond angles listed in the table below were found to deviate more than 4
sigma from standard bond angles (both standard valu

es and sigma for protein
residues have been taken from Engh and Huber [REF], for DNA/RNA from
Parkinson et al [REF]). In the table below for each strange angle the bond
angle and the number of standard deviations it differs from the standard
values is given. Please note that disulphide bridges are neglected. Atoms
starting with "-" belong to the previous residue in the sequence.

```
    4 GLU  (   4-) A  -   -C    N    CA  129.96
4.6
    5 GLN  (   5-) A  -   -C    N    CA  128.92
4.0
    6 GLN  (   6-) A  -   -C    N    CA  129.83
4.5
    8 ALA  (   8-) A  -   -C    N    CA  130.52
4.9
    9 PHE  (   9-) A  -   -C    N    CA  132.69
6.1
    9 PHE  (   9-) A  -    CB   CG   CD1 111.32   -
5.5
    9 PHE  (   9-) A  -    CG   CD1  CE1 131.15
6.1
    9 PHE  (   9-) A  -    CD2  CE2  CZ  104.96   -
8.4
    9 PHE  (   9-) A  -    CE1  CZ   CE2 106.68   -
7.4
    9 PHE  (   9-) A  -    CD1  CG   CD2 129.13
5.1
   11 GLU  (  11-) A  -   -C    N    CA  132.45
6.0
   12 ILE  (  12-) A  -    CA   C    O   112.13   -
5.1
   12 ILE  (  12-) A  -    N    CA   CB  119.16
5.1
   12 ILE  (  12-) A  -    C    CA   CB  117.91
4.1
   13 LEU  (  13-) A  -   -CA  -C    N   126.29
5.0
```

```
   13 LEU  (  13-) A  -   -C    N    CA  130.89
5.1
   14 HIS  (  14-) A  -   -C    N    CA  130.63
5.0
   15 LEU  (  15-) A  -   -C    N    CA  132.58
6.0
   16 PRO  (  16-) A  -   -CA  -C    N   126.00
6.1
   17 ASN  (  17-) A  -   -C    N    CA  130.72
5.0
   19 ASN  (  19-) A  -    CA   CB   CG  117.11
4.5
   21 GLU  (  21-) A  -   -C    N    CA  129.82
4.5
   22 GLN  (  22-) A  -   -C    N    CA  131.05
5.2
   23 ARG  (  23-) A  -   -C    N    CA  131.39
5.4
   24 ASN  (  24-) A  -   -C    N    CA  129.67
4.4
   25 GLY  (  25-) A  -   -C    N    CA  129.41
5.2
   26 PHE  (  26-) A  -   -C    N    CA  129.03
4.1
   26 PHE  (  26-) A  -    CA   CB   CG  119.53
5.7
   26 PHE  (  26-) A  -    CB   CG   CD1 113.74   -
4.1
   26 PHE  (  26-) A  -    CB   CG   CD2 113.60   -
4.2
   26 PHE  (  26-) A  -    CG   CD2  CE2 130.19
5.6
   26 PHE  (  26-) A  -    CD1  CE1  CZ  106.45   -
7.5
   26 PHE  (  26-) A  -    CE1  CZ   CE2 105.62   -
8.0
   26 PHE  (  26-) A  -    CD1  CG   CD2 127.99
4.4
   27 ILE  (  27-) A  -   -CA  -C    N   125.94
4.9
   28 GLN  (  28-) A  -   -C    N    CA  134.27
7.0
```

```
   29 SER  (  29-) A  -   -C    N    CA  130.08
4.7
   29 SER  (  29-) A  -    N    CA   CB  118.15
4.5
   32 ASP  (  32-) A  -   -C    N    CA  131.37
5.4
   34 PRO  (  34-) A  -   -CA  -C    N   123.21
4.2
   35 SER  (  35-) A  -   -C    N    CA  131.25
5.3
   36 GLN  (  36-) A  -   -C    N    CA  129.82
4.5
   38 ALA  (  38-) A  -   -C    N    CA  129.21
4.2
   39 ASN  (  39-) A  -   -C    N    CA  129.79
4.5
   40 LEU  (  40-) A  -   -C    N    CA  129.41
4.3
   41 LEU  (  41-) A  -   -C    N    CA  130.02
4.6
   42 ALA  (  42-) A  -   -C    N    CA  129.92
4.6
   43 GLU  (  43-) A  -   -C    N    CA  129.92
4.6
```

49 # Warning: High bond angle deviations
Bond angles were found to deviate more than normal from the mean standard
bond angles (normal values for protein residues were taken from Engh and
Huber [REF], for DNA/RNA from Parkinson et al [REF]). The RMS Z-score given
below is expected to be near 1.0 for a normally restrained data set, and
this is indeed observed for very high resolution X-ray structures. The fact
that it is higher than 2.0 in this structure might indicate that the
restraints used in the refinement were not strong enough. This will also
occur if a different bond angle dictionary is used.

RMS Z-score for bond angles: 2.136
RMS-deviation in bond angles: 3.884

50 # Error: Nomenclature error(s)
Checking for a hand-check. WHAT CHECK has over the course of this session
already corrected the handedness of atoms in several residues. These were
administrative corrections. These residues are listed here.

```
    4 GLU  (   4-) A  -
   11 GLU  (  11-) A  -
   20 GLU  (  20-) A  -
   21 GLU  (  21-) A  -
   23 ARG  (  23-) A  -
   43 GLU  (  43-) A  -
```

51 # Note: Chirality OK
All protein atoms have proper chirality. But, look at the previous table to
see a series of administrative chirality problems that were corrected
automatically upon reading-in the PDB file.

The average deviation= 1.805

52 # Note: Improper dihedral angle distribution OK
The RMS Z-score for all improper dihedrals in the structure is within normal
ranges.

Improper dihedral RMS Z-score : 1.643

53 # Error: Tau angle problems
The side chains of the residues listed in the table

below contain a tau
angle (N-Calpha-C) that was found to deviate from t
e expected value by
more than 4.0 times the expected standard deviation
. The number in the
table is the number of standard deviations this RMS
value deviates from
the expected value.

```
   13 LEU  (  13-) A  -   5.46
   30 LEU  (  30-) A  -   4.50
```

54 # Warning: High tau angle deviations
The RMS Z-score for the tau angles (N-Calpha-C) in
the structure is too
high. For well refined structures this number is ex
pected to be near 1.0.
The fact that it is higher than 1.5 worries us. How
ever, we determined the
tau normal distributions from 500 high-resolution X
-ray structures, rather
than from CSD data, so we cannot be 100 percent cer
tain about these numbers.

Tau angle RMS Z-score : 1.868

55 # Note: Side chain planarity OK
All of the side chains of residues that have an int
act planar group are
planar within expected RMS deviations.

56 # Error: Connections to aromatic rings out of
plane
The atoms listed in the table below are connected t
o a planar aromatic group
in the sidechain of a protein residue but were foun
d to deviate from the
least squares plane.

For all atoms that are connected to an aromatic sid
e chain in a protein
residue the distance of the atom to the least squar
es plane through the
aromatic system was determined. This value was divi
ded by the standard
deviation from a distribution of similar values fro
m a database of small
molecule structures.

```
   26 PHE  (  26-) A  -    CB  10.83
    9 PHE  (   9-) A  -    CB   8.30
```

Since there is no DNA and no protein with hydrogens
, no uncalibrated
planarity check was performed.

 Ramachandran Z-score : -1.839

57 # Note: Ramachandran Z-score OK
The score expressing how well the backbone conforma
tions of all residues
correspond to the known allowed areas in the Ramach
andran plot is within
expected ranges for well-refined structures.

 Ramachandran Z-score : -1.839

58 # Note: Torsion angles OK
All residues that are intact have normal overall to
rsion angle scores.

59 # Warning: Backbone evaluation reveals unusual
 conformations
The residues listed in the table below have abnorma
l backbone torsion
angles.

Residues with `forbidden' phi-psi combinations are

listed, as well as
residues with unusual omega angles (deviating by more than 3 sigma from the
normal value). Please note that it is normal if about 5 percent of the
residues is listed here as having unusual phi-psi combinations.

```
    8 ALA  (   8-) A  - omega poor
   10 TYR  (  10-) A  - omega poor
   11 GLU  (  11-) A  - omega poor
   12 ILE  (  12-) A  - Poor phi/psi
   13 LEU  (  13-) A  - Poor phi/psi
   17 ASN  (  17-) A  - Poor phi/psi
   22 GLN  (  22-) A  - omega poor
   25 GLY  (  25-) A  - omega poor
   27 ILE  (  27-) A  - omega poor
```

chi-1/chi-2 correlation Z-score : 1.808

60 # Note: chi-1/chi-2 angle correlation Z-score OK
The score expressing how well the chi-1/chi-2 angles of all residues
correspond to the populated areas in the database is
within expected ranges for well-refined structures.

chi-1/chi-2 correlation Z-score : 1.808

61 # Warning: Unusual rotamers
The residues listed in the table below have a rotamer that is not seen very
often in the database of solved protein structures. This option determines
for every residue the position specific chi-1 rotamer distribution.
Thereafter it verified whether the actual residue in the molecule has the
most preferred rotamer or not. If the actual rotame

r is the preferred one,
the score is 1.0. If the actual rotamer is unique, the score is 0.0. If
there are two preferred rotamers, with a population distribution of 3:2 and
your rotamer sits in the lesser populated rotamer, the score will be 0.667.
No value will be given if insufficient hits are found in the database.

It is not necessarily an error if a few residues have rotamer values below
0.3, but careful inspection of all residues with these low values could be
worth it.

```
   29 SER  (  29-) A  -   0.35
```

62 # Note: Backbone conformations OK
None of the residues have abnormal backbone conformations

63 # Note: Backbone conformation Z-score OK
The backbone conformation analysis gives a score that is normal for well
refined protein structures.

```
 Backbone conformation Z-score : 2.363
```

64 # Warning: Omega angle restraints not strong enough
The omega angles for trans-peptide bonds in a structure is expected to give
a gaussian distribution with the average around +178 degrees, and a standard
deviation around 5.5. In the current structure the standard deviation of
this distribution is above 7.0, which indicates that the omega values have

been under-restrained.

Omega average and std. deviation= 173.780 7.885

65 # Note: PRO puckering amplitude OK
Puckering amplitudes for all PRO residues are withi
n normal ranges.

66 # Warning: Unusual PRO puckering phases
The proline residues listed in the table below have
 a puckering phase that is
not expected to occur in protein structures. Pucker
ing parameters were
calculated by the method of Cremer and Pople [REF].
 Normal PRO rings
approximately show a so-called envelope conformatio
n with the C-gamma atom
above the plane of the ring (phi=+72 degrees), or a
 half-chair conformation
with C-gamma below and C-beta above the plane of th
e ring (phi=-90 degrees).
If phi deviates strongly from these values, this is
 indicative of a very
strange conformation for a PRO residue, and definit
ely requires a manual
check of the data. Be aware that this is a warning
with a low confidence
level. See: Who checks the checkers? Four validatio
n tools applied to eight
atomic resolution structures [REF].

```
   34 PRO  (  34-) A  - -113.7 envelop C-gamma (-10
8 degrees)
```

67 # Note: Backbone oxygen evaluation OK
All residues for which the local backbone conformat
ion could be found in the
WHAT CHECK database have a normal backbone oxygen p
osition.

```
DBG> IAA out of range in WIFIRA 44 43 43
                     10        20        30
40
                      |         |         |
 |
    1 -   43 FNKEQQNAFYEILHLPNLNEEQRNGFIQSLKDDPSQSA
NLLAE
(   1)-(  43)  HHHHHHHHHHHTTTT  HHHHHHHHTHHHH HHHHH
HHHH
```

ERROR. File not found:
/dssp//hom.DSSP
ERROR. File could not be opened. Disk full? Directo
ry?

DSSP file not yet present in database. Made it...
Things are going very wrong
ERROR in internal write in routine PDBW09. This cou
ld be a bug.

68 # Note: Peptide bond conformations
There was no need to complain about a single amino
acid

DBG> Symmetry matrices are locked. SYMRDU aborted.

69 # Error: Abnormally short interatomic distance
s
The pairs of atoms listed in the table below have a
n unusually short
interactomic distance; each bump is listed in only
one direction.

The contact distances of all atom pairs have been c
hecked. Two atoms are
said to `bump' if they are closer than the sum of t
heir Van der Waals radii

minus 0.40 Angstrom. For hydrogen bonded pairs a tolerance of 0.55 Angstrom
is used. The first number in the table tells you how much shorter that
specific contact is than the acceptable limit. The second distance is the
distance between the centres of the two atoms. Although we believe that two
water atoms at 2.4 A distance are too close, we only report water pairs that
are closer than this rather short distance.

The last text-item on each line represents the status of the atom pair. If
the final column contains the text 'HB', the bump criterion was relaxed
because there could be a hydrogen bond. Similarly relaxed criteria are used
for 1-3 and 1-4 interactions (listed as 'B2' and 'B3', respectively).
If the last column is 'BF', the sum of the B-factors of the atoms is higher
than 80, which makes the appearance of the bump somewhat less severe because
the atoms probably are not there anyway. BL, on the other hand, indicates
that the bumping atoms both have a low B-factor, and that makes the bumps
more worrisome. INTRA and INTER indicate whether the clashes are between
atoms in the same asymmetric unit, or atoms in symmetry related asymmetric
units, respectively.

Bumps between atoms for which the sum of their occupancies is lower than one
are not reported. If the MODEL number does not exist (as is the case in most
X-ray files), a minus sign is printed instead.

```
    9 PHE  (   9-) A  -    CZ  <->    26 PHE  (  26-) A  -    CZ
```

1.03 2.17 INTRA BL
9 PHE (9-) A - CE2 <-> 26 PHE (26
-) A - CE1
1.03 2.17 INTRA BL
9 PHE (9-) A - CE2 <-> 26 PHE (26
-) A - CZ
1.03 2.17 INTRA BL
9 PHE (9-) A - CZ <-> 26 PHE (26
-) A - CE1
1.02 2.18 INTRA BL
9 PHE (9-) A - CZ <-> 26 PHE (26
-) A - CD1
0.83 2.37 INTRA BL
9 PHE (9-) A - CE2 <-> 26 PHE (26
-) A - CE2
0.81 2.39 INTRA BL
9 PHE (9-) A - CD2 <-> 26 PHE (26
-) A - CE1
0.81 2.39 INTRA BL
9 PHE (9-) A - CD2 <-> 26 PHE (26
-) A - CZ
0.81 2.39 INTRA BL
9 PHE (9-) A - CE2 <-> 26 PHE (26
-) A - CD1
0.81 2.39 INTRA BL
9 PHE (9-) A - CE1 <-> 26 PHE (26
-) A - CE1
0.81 2.39 INTRA BL
9 PHE (9-) A - CZ <-> 26 PHE (26
-) A - CE2
0.80 2.40 INTRA BL
9 PHE (9-) A - CD1 <-> 26 PHE (26
-) A - CE1
0.78 2.42 INTRA BL
9 PHE (9-) A - CE1 <-> 26 PHE (26
-) A - CZ
0.78 2.42 INTRA BL
9 PHE (9-) A - CZ <-> 26 PHE (26
-) A - CG
0.74 2.46 INTRA BL
9 PHE (9-) A - CG <-> 26 PHE (26
-) A - CZ

```
0.73    2.47  INTRA BL
    9 PHE  (   9-) A  -    CE2 <->    26 PHE  (  26
-) A  -    CD2
0.73    2.47  INTRA BL
    9 PHE  (   9-) A  -    CG  <->    26 PHE  (  26
-) A  -    CE1
0.73    2.47  INTRA BL
    9 PHE  (   9-) A  -    CZ  <->    26 PHE  (  26
-) A  -    CD2
0.72    2.48  INTRA BL
    9 PHE  (   9-) A  -    CD1 <->    26 PHE  (  26
-) A  -    CZ
0.69    2.51  INTRA BL
    9 PHE  (   9-) A  -    CE2 <->    26 PHE  (  26
-) A  -    CG
0.69    2.51  INTRA BL
   26 PHE  (  26-) A  -    O   <->    29 SER  (  29
-) A  -    N
0.04    2.66  INTRA BL


# 70 # Note: Some notes regarding these bumps
The bumps have been binned in 5 categories ranging
from 'should deal with'
till 'must fix'. Additionally, the integrated sum o
f all bumps, the squared
sum of all bumps, and these latter two values norma
lized by the number of
contacts are listed too for comparison purposes bet
ween, for example, small
and large proteins.

Total bump value: 16.422
Total bump value per residue: 0.488
Total number of bumps: 21
Total squared bump value: 13.666
Total number of bumps in the mildest bin: 1
Total number of bumps in the second bin: 0
Total number of bumps in the middle bin: 7
Total number of bumps in the fourth bin: 9
Total number of bumps in the worst bin: 4
```

71 # Note: Inside/Outside residue distribution no
rmal
The distribution of residue types over the inside a
nd the outside of the
protein is normal.

inside/outside RMS Z-score : 1.006

72 # Note: Inside/Outside RMS Z-score plot
The Inside/Outside distribution normality RMS Z-sco
re over a 15 residue
window is plotted as function of the residue number
. High areas in the plot
(above 1.5) indicate unusual inside/outside pattern
s.

In the TeX file, a plot has been inserted here

Chain identifier: A

73 # Note: Packing environment OK
None of the individual amino acid residues has a ba
d packing environment.

74 # Note: No series of residues with bad packing
environment
There are no stretches of three or more residues ea
ch having a packing score
worse than -4.0.

75 # Note: Structural average packing environment
OK
The structural average packing score is within norm
al ranges.

Average for range 1 - 43 : -1.258

76 # Note: Quality value plot
The quality value smoothed over a 10 residue window
 is plotted as function
of the residue number. Low areas in the plot (below
 -2.0) indicate unusual
packing.

In the TeX file, a plot has been inserted here

 Chain identifier: A

```
 All   contacts    : Average =  0.031 Z-score =   0
.14
 BB-BB contacts    : Average =  0.038 Z-score =   0
.14
 BB-SC contacts    : Average = -0.005 Z-score =  -0
.12
 SC-BB contacts    : Average = -0.008 Z-score =   0
.06
 SC-SC contacts    : Average = -0.113 Z-score =  -0
.47
```

77 # Note: Second generation packing environment
OK
None of the individual amino acid residues has a ba
d packing environment.

78 # Note: No series of residues with abnormal ne
w packing environment
There are no stretches of four or more residues eac
h having a packing
Z-score worse than -1.75.

79 # Note: Structural average packing Z-score OK
The structural average for the second generation pa
cking score is within
normal ranges.

```
 All   contacts    : Average =  0.031 Z-score =   0
.14
 BB-BB contacts    : Average =  0.038 Z-score =   0
.14
 BB-SC contacts    : Average = -0.005 Z-score =  -0
.12
 SC-BB contacts    : Average = -0.008 Z-score =   0
.06
 SC-SC contacts    : Average = -0.113 Z-score =  -0
.47

DBG> IAA out of range in WIFIRA 44 43 43

# 80 # Note: Second generation quality Z-score plot

The second generation quality Z-score smoothed over
 a 10 residue window
is plotted as function of the residue number. Low a
reas in the plot (below
-1.3) indicate unusual packing.

In the TeX file, a plot has been inserted here

 Chain identifier: A

DBG> Symmetry matrices are locked. SYMRDU aborted.
DBG> FLPUNT opened at unit 77
Number of ambiguities touching ambiguities: 0

# 81 # Error: His, Asn, Gln side chain flips
Listed here are Histidine, Asparagine or Glutamine
residues for
which the orientation determined from hydrogen bond
ing analysis are
different from the assignment given in the input. E
ither they could
form energetically more favourable hydrogen bonds i
f the terminal
group was rotated by 180 degrees, or there is no as
signment in the
input file (atom type 'A') but an assignment could
be made. Be aware,
```

though, that if the topology could not be determine d for one or more
ligands, then this option will make errors.

```
   22 GLN  (  22-) A  -
```

82 # Note: Histidine type assignments
For all complete HIS residues in the structure a te ntative assignment to
HIS-D (protonated on ND1), HIS-E (protonated on NE2), or HIS-H (protonated
on both ND1 and NE2, positively charged) is made ba sed on the hydrogen bond
network. A second assignment is made based on which of the Engh and Huber
[REF] histidine geometries fits best to the structu re.

In the table below all normal histidine residues ar e listed. The assignment
based on the geometry of the residue is listed firs t, together with the RMS
Z-score for the fit to the Engh and Huber parameter s. For all residues where
the H-bond assignment is different, the assignment is listed in the last
columns, together with its RMS Z-score to the Engh and Huber parameters.

As always, the RMS Z-scores should be close to 1.0 if the residues were
restrained to the Engh and Huber parameters during refinement, and if
enough (high resolution) data is available.

Please note that because the differences between th e geometries of the
different types are small it is possible that the g eometric assignment given
here does not correspond to the type used in refine ment. This is especially

true if the RMS Z-scores are much higher than 1.0.

If the two assignments differ, or the `geometry' RM
S Z-score is high, it is
advisable to verify the hydrogen bond assignment, c
heck the HIS type used
during the refinement and possibly adjust it.

```
   14 HIS  (  14-) A  -   HIS-E   0.52
```

83 # Warning: Buried unsatisfied hydrogen bond do
nors
The buried hydrogen bond donors listed in the table
 below have a hydrogen
atom that is not involved in a hydrogen bond in the
 optimized hydrogen bond
network.

Hydrogen bond donors that are buried inside the pro
tein normally use all of
their hydrogens to form hydrogen bonds within the p
rotein. If there are any
non hydrogen bonded buried hydrogen bond donors in
the structure they will
be listed here. In very good structures the number
of listed atoms will tend
to zero.

Waters are not listed by this option.

```
    6 GLN  (   6-) A  -    N
    7 ASN  (   7-) A  -    N
    9 PHE  (   9-) A  -    N
   30 LEU  (  30-) A  -    N
   35 SER  (  35-) A  -    N
   37 SER  (  37-) A  -    OG
```

84 # Note: Buried hydrogen bond acceptors OK
All buried polar side-chain hydrogen bond acceptors
 are involved in a

hydrogen bond in the optimized hydrogen bond networ
k.

85 # Note: Some notes regarding these donors and acceptors
The donors and acceptors have been counted, also as function of their
accessibility. The buried donors and acceptors have been binned in five
categories ranging from not forming any hydrogen bond till forming a poor
till perfect hydrogen bond. Obviously, the buried donors and acceptors
with no or just a poor hydrogen bond should be a topic of concern.

86 # Warning: No crystallisation information
No, or very inadequate, crystallisation information was observed upon
reading the PDB file header records. This information should be available
in the form of a series of REMARK 280 lines. Without this information a
few things, such as checking ions in the structure, cannot be performed
optimally.

87 # Note: No ions (of a type we can validate) in structure
Since there are no ions in the structure of a type we can validate, this
check will not be executed.

Since there are no waters, the water check has been skipped.

88 # Note: Content of the PDB file as interpreted by WHAT CHECK
Content of the PDB file as interpreted by WHAT CHECK.
WHAT CHECK has read your PDB file, and stored it internally in what is called
'the soup'. The content of this soup is listed here. An extensive explanation
of all frequently used WHAT CHECK output formats can be found at
swift.cmbi.ru.nl. Look under output formats. A course on reading this
'Molecules' table is part of the WHAT CHECK website.

```
     1     1 (    1)    43 (   43) A Protein             checkset
```

89 # Note: Summary report
This is an overall summary of the quality of the structure as compared with
current reliable structures. Numbers in brackets are the average and standard
deviation observed for a large number of files determined with a similar
resolution.

The second table mostly gives an impression of how well the model conforms
to common refinement restraint values.

```
 Structure Z-scores, positive is better than average:
  Resolution read from PDB file  :  -1.000
  1st generation packing quality :  -1.896
  2nd generation packing quality :   0.139
  Ramachandran plot appearance   :  -1.839
  chi-1/chi-2 rotamer normality  :   1.808
  Backbone conformation          :   2.363
  Inside/Outside distribution    :   1.006
```

```
 RMS Z-scores, should be close to 1.0:
  Bond lengths                       :   1.762 (loose)
  Bond angles                        :   2.136 (loose)
  Omega angle restraints             :   1.434 (loose)
  Side chain planarity               :   0.870
  Improper dihedral distribution :   1.643 (loose)
  B-factor distribution              :   0.387
```

==============

WHAT IF
    G.Vriend,
      WHAT IF: a molecular modelling and drug desig
n program,
    J. Mol. Graph. 8, 52--56 (1990).

WHAT_CHECK (verification routines from WHAT IF)
    R.W.W.Hooft, G.Vriend, C.Sander and E.E.Abola,
      Errors in protein structures
    Nature 381, 272 (1996).
    (see also http://swift.cmbi.ru.nl/gv/whatcheck
for a course and extra
    information)

Bond lengths and angles, protein residues
    R.Engh and R.Huber,
      Accurate bond and angle parameters for X-ray
protein structure
      refinement,
    Acta Crystallogr. A47, 392--400 (1991).

Bond lengths and angles, DNA/RNA
    G.Parkinson, J.Voitechovsky, L.Clowney, A.T.Bru
enger and H.Berman,
      New parameters for the refinement of nucleic
acid-containing structures
    Acta Crystallogr. D52, 57--64 (1996).

DSSP
    W.Kabsch and C.Sander,
      Dictionary of protein secondary structure: pa

ttern
recognition of hydrogen bond and geometrical features
Biopolymers 22, 2577--2637 (1983).

Hydrogen bond networks
R.W.W.Hooft, C.Sander and G.Vriend,
Positioning hydrogen atoms by optimizing hydrogen bond networks in
protein structures
PROTEINS, 26, 363--376 (1996).

Matthews' Coefficient
B.W.Matthews
Solvent content of Protein Crystals
J. Mol. Biol. 33, 491--497 (1968).

Protein side chain planarity
R.W.W. Hooft, C. Sander and G. Vriend,
Verification of protein structures: side-chain planarity
J. Appl. Cryst. 29, 714--716 (1996).

Puckering parameters
D.Cremer and J.A.Pople,
A general definition of ring puckering coordinates
J. Am. Chem. Soc. 97, 1354--1358 (1975).

Quality Control
G.Vriend and C.Sander,
Quality control of protein models: directional atomic
contact analysis,
J. Appl. Cryst. 26, 47--60 (1993).

Ramachandran plot
G.N.Ramachandran, C.Ramakrishnan and V.Sasisekharan,
Stereochemistry of Polypeptide Chain Conformations
J. Mol. Biol. 7, 95--99 (1963).

R.W.W. Hooft, C.Sander and G.Vriend,
Objectively judging the quality of a protein structure from a
Ramachandran plot
CABIOS (1997), 13, 425--430.

Symmetry Checks
R.W.W.Hooft, C.Sander and G.Vriend,
Reconstruction of symmetry related molecules from protein
data bank (PDB) files
J. Appl. Cryst. 27, 1006--1009 (1994).

Tau angle
W.G.Touw and G.Vriend
On the complexity of Engh and Huber refinement restraints: the angle
tau as example.
Acta Crystallogr D 66, 1341--1350 (2010).

Ion Checks
I.D.Brown and K.K.Wu,
Empirical Parameters for Calculating Cation-Oxygen Bond Valences
Acta Cryst. B32, 1957--1959 (1975).

M.Nayal and E.Di Cera,
Valence Screening of Water in Protein Crystals Reveals Potential Na+
Binding Sites
J.Mol.Biol. 256 228--234 (1996).

P.Mueller, S.Koepke and G.M.Sheldrick,
Is the bond-valence method able to identify metal atoms in protein
structures?
Acta Cryst. D 59 32--37 (2003).

Checking checks
K.Wilson, C.Sander, R.W.W.Hooft, G.Vriend, et al.
Who checks the checkers

J.Mol.Biol. (1998) 276,417-436.
/home/vriend/whatif/dbdata/pdbout2html
After running WHAT IF's WHAT CHECK option many things have happened to
the data structure that might not be optimal for many other options.
FULCHK therefore is a so-called terminal option, i.e. after running
the validation option, WHAT IF will restart without any coordinates
in the soup; so the molecule you just checked got deleted together
with anything else you might have had in the SOUP.
==============

WHAT IF
G.Vriend,
WHAT IF: a molecular modelling and drug design program,
J. Mol. Graph. 8, 52--56 (1990).

WHAT_CHECK (verification routines from WHAT IF)
R.W.W.Hooft, G.Vriend, C.Sander and E.E.Abola,
Errors in protein structures
Nature 381, 272 (1996).
(see also http://swift.cmbi.ru.nl/gv/whatcheck for a course and extra
information)

Bond lengths and angles, protein residues
R.Engh and R.Huber,
Accurate bond and angle parameters for X-ray protein structure
refinement,
Acta Crystallogr. A47, 392--400 (1991).

Bond lengths and angles, DNA/RNA
G.Parkinson, J.Voitechovsky, L.Clowney, A.T.Bruenger and H.Berman,
New parameters for the refinement of nucleic acid-containing structures

Acta Crystallogr. D52, 57--64 (1996).

DSSP
W.Kabsch and C.Sander,
Dictionary of protein secondary structure: pa
ttern
recognition of hydrogen bond and geometrical
features
Biopolymers 22, 2577--2637 (1983).

Hydrogen bond networks
R.W.W.Hooft, C.Sander and G.Vriend,
Positioning hydrogen atoms by optimizing hydr
ogen bond networks in
protein structures
PROTEINS, 26, 363--376 (1996).

Matthews' Coefficient
B.W.Matthews
Solvent content of Protein Crystals
J. Mol. Biol. 33, 491--497 (1968).

Protein side chain planarity
R.W.W. Hooft, C. Sander and G. Vriend,
Verification of protein structures: side-chai
n planarity
J. Appl. Cryst. 29, 714--716 (1996).

Puckering parameters
D.Cremer and J.A.Pople,
A general definition of ring puckering coordi
nates
J. Am. Chem. Soc. 97, 1354--1358 (1975).

Quality Control
G.Vriend and C.Sander,
Quality control of protein models: directiona
l atomic
contact analysis,
J. Appl. Cryst. 26, 47--60 (1993).

Ramachandran plot

G.N.Ramachandran, C.Ramakrishnan and V.Sasisekharan,
Stereochemistry of Polypeptide Chain Conformations
J. Mol. Biol. 7, 95--99 (1963).
R.W.W. Hooft, C.Sander and G.Vriend,
Objectively judging the quality of a protein structure from a
Ramachandran plot
CABIOS (1997), 13, 425--430.

Symmetry Checks
R.W.W.Hooft, C.Sander and G.Vriend,
Reconstruction of symmetry related molecules from protein
data bank (PDB) files
J. Appl. Cryst. 27, 1006--1009 (1994).

Tau angle
W.G.Touw and G.Vriend
On the complexity of Engh and Huber refinement restraints: the angle
tau as example.
Acta Crystallogr D 66, 1341--1350 (2010).

Ion Checks
I.D.Brown and K.K.Wu,
Empirical Parameters for Calculating Cation-Oxygen Bond Valences
Acta Cryst. B32, 1957--1959 (1975).

M.Nayal and E.Di Cera,
Valence Screening of Water in Protein Crystals Reveals Potential Na+
Binding Sites
J.Mol.Biol. 256 228--234 (1996).

P.Mueller, S.Koepke and G.M.Sheldrick,
Is the bond-valence method able to identify metal atoms in protein
structures?
Acta Cryst. D 59 32--37 (2003).

Checking checks
K.Wilson, C.Sander, R.W.W.Hooft, G.Vriend, et a
l.
Who checks the checkers
J.Mol.Biol. (1998) 276,417-436.
