# Supplementary material for: Protein structure quality assessment based on the distance profiles of consecutive backbone Cα atoms
Source: F1000Res. 2013 Dec 17;2:211. Originally published 2013 Oct 10. [Version 3] doi: 10.12688/f1000research.2-211.v3 (PMC3892923; doi:10.12688/f1000research.2-211.v3)
Supplement: Peer review history

# Peer review: Referee response for version 3

**DOI:** 10.5256/f1000research.3328.r4038

**Published:** 2014-03-11

**Authors:** Bairong Shen

**Affiliations:** 1 Center for Systems Biology, Soochow University, Suzhou, China

I have read this submission. I believe that I have an appropriate level of expertise to confirm that it is of an acceptable scientific standard.

# Peer review: Referee response for version 3

**DOI:** 10.5256/f1000research.3328.r3986

**Published:** 2014-03-05

**Authors:** Simon Lovell

**Affiliations:** 1 Faculty of Life Sciences, University of Manchester, Manchester, UK

I have read this submission. I believe that I have an appropriate level of expertise to confirm that it is of an acceptable scientific standard.

# Peer review: Referee response for version 1

**DOI:** 10.5256/f1000research.2356.r2086

**Published:** 2013-11-06

**Authors:** Simon Lovell

**Affiliations:** 1 Faculty of Life Sciences, University of Manchester, Manchester, UK

This is a nice, straightforward analysis of features of protein decoy sets. The authors find that a simple, novel measure of protein geometry is sufficient to distinguish native structures from decoys in the popular fisa decoy set. A rational response to this work would be to include measures of geometry such as that which are used here in the generation of the decoys. This would make the measure less useful, but would improve decoy sets.

I have read this submission. I believe that I have an appropriate level of expertise to confirm that it is of an acceptable scientific standard.

# Response

**Published:** 2013-11-18

**Authors:** Sandeep Chakraborty

**Affiliations:** Tata Institute of Fundamental Research, India

Dear Dr Lovell,

Thank you for your encouraging comments on our manuscript. We took a lot of inspiration from your work "Artefacts and biases affecting the evaluation of scoring functions on decoy sets for protein structure prediction. *Bioinformatics.* 2009" (ref 52), while writing our manuscript.

Additionally, we have revised our manuscript adding some additional results we have obtained on the CASP8 I-TASSER decoy set.

Warm regards,

Sandeep

# Peer review: Referee response for version 1

**DOI:** 10.5256/f1000research.2356.r2091

**Published:** 2013-10-30

**Authors:** Bairong Shen

**Affiliations:** 1 Center for Systems Biology, Soochow University, Suzhou, China

This paper proposes an absolute discriminator to identify non-native protein structures based on backbone C _α_ atom distance. Interestingly the authors found that most methods performed poorly in the fisa decoy set from the Decoys “R” Us database and remind researchers to be cautious of using this decoy set since most of the sub-structures in the fisa decoy set are incorrect. This work provides a simple and fast assessment for protein structure quality.

I have read this submission. I believe that I have an appropriate level of expertise to confirm that it is of an acceptable scientific standard.

# Response

**Published:** 2013-11-18

**Authors:** Sandeep Chakraborty

**Affiliations:** Tata Institute of Fundamental Research, India

Dear Dr Shen,

Thank you for taking the time to review our manuscript. We appreciate your encouraging comments. We have revised our manuscript with some additional results from the CASP8 I-TASSER decoy set, and also cited some recent manuscripts which had been published since the version 1 went online. We hope you find the revised version improved.

Warm regards,

Sandeep
